# The Divergence History of Two Japanese *Torreya* Taxa (Taxaceae): Implications for Species Diversification in the Japanese Archipelago

**DOI:** 10.3390/plants14101537

**Published:** 2025-05-20

**Authors:** Qian Ou, Xin Huang, Dingguo Pan, Shulan Wang, Yuting Huang, Sisi Lu, Yujin Wang, Yixuan Kou

**Affiliations:** 1 Key Laboratory of Ecology of Rare and Endangered Species and Environmental Protection, Ministry of Education, Guangxi Normal University, Guilin 541006, China; ouqian0711@163.com (Q.O.); 19580780849@163.com (X.H.); 14780048035@163.com (D.P.); wangshulan_1003@163.com (S.W.); skhuangyuting26@163.com (Y.H.); leleharibo@163.com (S.L.); 2 Guangxi Key Laboratory of Landscape Resources Conservation and Sustainable Utilization in Lijiang River Basin, Guangxi Normal University, Guilin 541006, China; 3 University Engineering Research Center of Bioinformation and Genetic Improvement of Specialty Crops, Guilin 541006, China; 4 State Key Laboratory of Grassland Agro-Ecosystems, School of Life Sciences, Lanzhou University, Lanzhou 730000, China

**Keywords:** vicariant speciation, ecological differentiation, Quaternary, continental islands, *Torreya nucifera*

## Abstract

The Japanese archipelago as a continental island of the Eurasia continent and harboring high levels of plant species diversity provides an ideal geographical setting for investigating vicariant allopatric speciation due to the sea-level fluctuations associated with climatic oscillations during the Quaternary. In this study, three chloroplast DNA regions and 14 nuclear loci were sequenced for 31 individuals from three populations of *Torreya nucifera* var. *nucifera* and 52 individuals from three populations of *T. nucifera* var. *radicans*. Population genetic analyses (Network, STRUCTURE and phylogeny) revealed that the genetic boundaries of the two varieties are distinct, with high genetic differentiation (*F*_ST_) of 0.9619 in chloroplast DNA and 0.6543 in nuclear loci. The relatively ancient divergence times between the two varieties were estimated to 3.03 Ma by DIYABC and 1.77 Ma by IMa2 when dated back to the late Pliocene and the early Pleistocene, respectively. The extremely weak gene flow (*2Nm* = 0.1) between the two varieties was detected by IMa2, which might be caused by their population expansion since the early Pleistocene (~2.0 Ma) inferred in the Bayesian skyline plots and DIYABC. Niche modeling showed that the two varieties had significant ecological differentiation (*p* < 0.001) since the Last Interglacial even earlier. These results demonstrate that vicariant allopatric speciation due to sea-level fluctuations may be a common mode of speciation in the Japanese archipelago. This finding provides insights into the understanding of species diversification in the Japanese Archipelago and even East Asian flora under climatic oscillations during the Quaternary.

## 1. Introduction

Climate oscillations since the Quaternary have had profound consequences for species diversification on islands [1,2]. Moreover, this impact appears to be more pronounced on continental islands, where the sea-level fluctuations associated with climatic oscillations have repeatedly severed and reconnected these islands with the adjacent mainland (e.g., [3,4]). Such recurrent geographic changes create conditions conducive to vicariant allopatric speciation, consequently leading to the assembly of closely related species on continental islands [5,6]. However, comparable biogeographic patterns of species diversity could also be shaped through in situ speciation on continental islands, provided that the geological and environmental heterogeneity on these islands enables populations to differentiate following localized adaptation and refugial isolation (e.g., the Japanese archipelago [7,8]). Thus, inferring the divergence history of closely related taxa could assist in understanding the geographic modes of speciation underlying species diversification on continental islands [9,10]. In addition, given that islands are typically limited in size, localized ecological differences are essential to maintaining species boundaries, especially for closely related species recently formed through any geographic modes of speciation [5,11].

The Japanese archipelago, located at the eastern edge of the Eurasia continent, harbors high levels of plant species diversity as a critically important part of the East Asian flora [12,13]. It has been uncovered that climate oscillations during the late Quaternary, especially glacial–interglacial cycles, resulted in genetic differentiation and hence potential speciation in many plant taxa (in situ lineage and species diversification) [6,14]. For example, the genetic divergence occurred between northern and central (as refugia during interglacial periods) Japan due to repeated range expansion and contraction [8], and occurred between the Pacific Ocean side and Japan Sea side of the islands due to climatic differences caused by the Asian monsoon and the Tsushima Current [7,15]. However, the fluctuations in sea level, with frequent shifts between high and low levels during the past 10 Ma, and particularly during the Quaternary, have alternately separated and joined Japan with the Eurasia continent [16,17,18] and consequently created conditions for vicariant allopatric differentiation and speciation. This is probably why many closely related species pairs endemic in Japan could be found with distinct genetic differentiation and morphological differences [19], such as *Fagus crenata* Blume and *F. japonica* Maxim. that are both endemic in Japan but probably originated allopatrically from different ancestors located in China [20]. Thus, vicariant allopatric speciation may be a common mode of speciation that occurred under climatic oscillations during the Quaternary or even earlier, and potentially contributed to the present-day species diversification in the Japanese archipelago.

**Figure 1:**
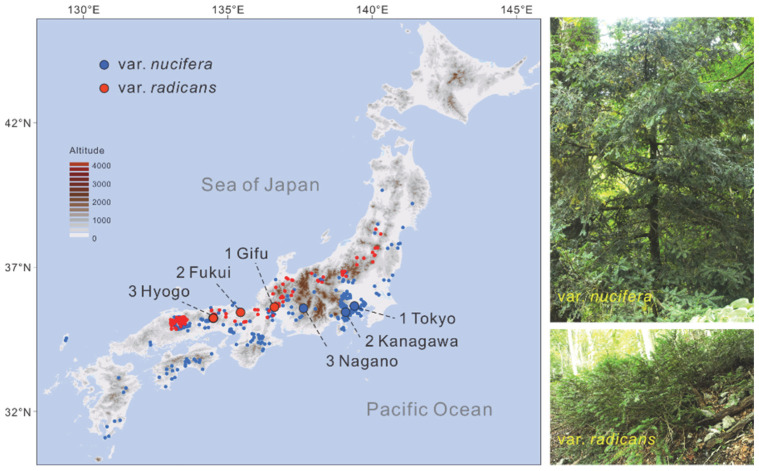
The distribution of six populations sampled in the study and occurrence records obtained from the Global Biodiversity Information Facility (GBIF) and the morphological photographs of *Torreya nucifera* var. *nucifera* and *T. nucifera* var. *radicans*.

*Torreya nucifera* (L.) Siebold et Zucc. (Taxaceae) provides an ideal study system for elucidating the processes of speciation in the Japanese Archipelago under historical climatic oscillations. Although two infraspecific taxa, *T. nucifera* var. *nucifera* and *T. nucifera* var. *radicans* Nakai, are commonly recognized under this species [21], there have been some taxonomic disputes. *T*. *nucifera* var. *radicans* was once raised to species level as a separate species *T. fruticosa* Nakai [22] and after a long interval treated as a synonym of *T. nucifera* [23,24]. Moreover, Aizawa and Worth [19] suggested resuming the use of *T. fruticosa* instead of *T. nucifera* var. *radicans* based on the evidence from phylogenetic analyses. Considering the limitation of the data employed in the phylogenetic analyses, we provisionally retain the commonly used name *T. nucifera* var. *radicans* in the present study. However, in the phylogenetic study of *Torreya*, *T. nucifera* var. *radicans* was revealed that is more closely related to *T. grandis* Fortune ex Lindl. endemic in China than to *T. nucifera* var. *nucifera* in chloroplast phylogenetic tree, while its haplotypes of nuclear ITS sequences are separately grouped with *T. grandis* and *T. nucifera* var. *nucifera*, but received low branch support [19]. Also, the two Japanese Torreya taxa are apparently distinct morphologically, namely in that *T. nucifera* var. *nucifera* shows an erect tree form with a single stem, but *T*. *nucifera* var. *radicans* shows stunted shrub form with multiple creeping or decumbent stems ([19]; Figure 1). These clues imply that the two varieties might have speciated allopatrically from a different most recent common ancestor. In addition, the geographic distributions of *T. nucifera* var. *nucifera* and *T. nucifera* var. *radicans* are mainly restricted on the Pacific Ocean side and the Japan Sea side of the Japanese archipelago, respectively (Figure 1). Notably, the climate on both sides is in marked contrast, a dry climate in winter on the Pacific Ocean side and heavy snowfall during winter on the Japan Sea side [7,15,19]. These contrasting climatic conditions might have facilitated adaptive divergence and/or maintained species boundary of the two Japanese *Torreya* taxa.

While most previous studies in plant diversifications have focused on intraspecific variations in the Japanese Archipelago [7,8], but studies on infraspecific or interspecific divergence history under a more prolonged temporal framework are very rare [6]. Here, we combined multilocus-based genetic approaches with ecological niche analyses to elucidate the divergence history of *T. nucifera* var. *nucifera* and *T. nucifera* var. *radicans* and determine the role of climatic variables in separation of the two taxa. Our specific objectives were to ascertain the following: What is the extent of genetic differentiation between the two taxa? What is the geographic mode of speciation of the two taxa, in situ or allopatric origins? What role do climatic factors play in the separation of the two varieties? The outcome of these analyses will contribute to a better understanding of species diversification in the Japanese Archipelago under climatic changes during the Quaternary.

## 2. Results

### 2.1. Genetic Diversity and Neutrality Tests

Eight cpDNA haplotypes were identified based on seven indels and twelve substitutions across three chloroplast regions, *rpl16* (911 bp), *rpoB-trnC* (692 bp), and *trnL-trnF* (903 bp) (Table S1). The total haplotype diversity (*H*_d_) was slightly higher in *T. nucifera* var. *nucifera* (0.5935) than in *T. nucifera* var. *radicans* (0.3024), whereas nucleotide diversity (π) showed comparable values between the two varieties (0.00008 vs. 0.00013, respectively) (Table S1). Both varieties exhibited negative values for Tajima’s *D* and Fu’s *F*_S_ statistics; however, the values for *T. nucifera* var. *radicans* (−0.5176 and −0.528, respectively) were significantly closer to zero compared with those of *T. nucifera* var. *nucifera* (−1.7308 and −3.436, respectively).

The sequences of fourteen low-copy nuclear loci were aligned across all samples of *T. nucifera*, with a total length of 6075 bp. The level of genetic diversity in *T. nucifera* var. *nucifera* was slightly lower than that of *T. nucifera* var. *radicans*. A total of 44 segregating sites (*S*) were detected in 14 nuclear loci in *T. nucifera* var. *nucifera* compared to 72 in *T. nucifera* var. *radicans*. The numbers of haplotypes (*N*_h_) within populations ranged from 1 to 8 (mean = 3.00) in the former and from 1 to 15 (mean = 5.00) in the latter. The nucleotide diversity (*π*), Watterson’s parameter (*θ*_w_), and haplotype diversity (*H*_d_) in *T. nucifera* var. *nucifera* (0.00161, 0.00139 and 0.280, respectively) were consistently lower than those in *T. nucifera* var. *radicans* (0.00235, 0.00198 and 0.457 on average, respectively) (Table S2). Overall, genetic diversity analyses of nuclear loci and chloroplast regions consistently imply that the two Japanese *Torreya* taxa are likely to have experienced similar recent demographic histories.

The majority of nuclear loci did not deviate from the neutral expectation in three neutrality tests, Tajima’s *D*, Fu and Li’s D* and F* (Table S2). Four loci, *T147* in *T. nucifera* var. *nucifera*, and *T147*, *T203* and *T249* in *T. nucifera* var. *radicans*, showed a significant level of deviation. However, the significant signals for these loci were detected in only one of the three neutrality tests. For example, the locus *T147* in *T. nucifera* var. *nucifera* exhibited a significant signal in Tajima’s *D* test but showed no significant signals in Fu and Li’s D* and F* tests (Table S2). Therefore, all loci in this study were consistent with neutral evolution and were thus used for inferring demographic history.

### 2.2. Population Genetic Structure

**Figure 2:**
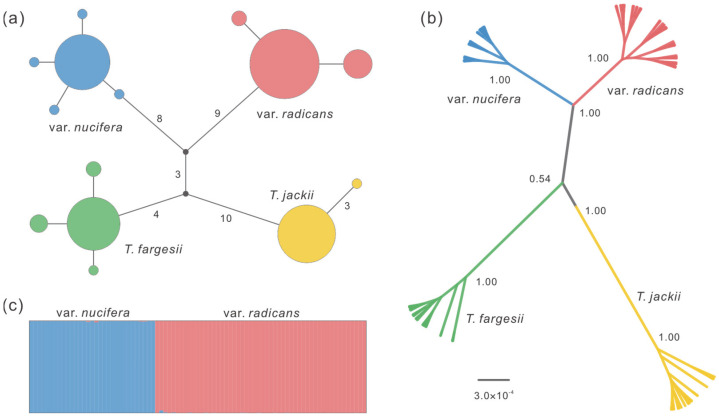
Population genetic structure of *T. nucifera* var. *nucifera* and *T. nucifera* var. *radicans*. (**a**) Chloroplast haplotype network of the two varieties and outgroups (*T. fargesii* and *T. jackii*). The sizes of the circles are proportional to the haplotype frequencies. Mutation steps greater than one are labeled with corresponding numeric values. (**b**) Phylogenetic relationships of the two varieties and outgroups inferred using BEAST based on 14 nuclear loci. The numbers on the branches represent support values. (**c**) STRUCTURE plot inferred for the two varieties under the optimal *K* value (*K* = 2).

The median-joining network of chloroplast haplotypes revealed substantial genetic differentiation between *T. nucifera* var. *nucifera* and *T. nucifera* var. *radicans*. Two distinct haplotype groups (H1–H5 and H6–H8) were exclusively fixed in *T. nucifera* var. *nucifera* and *T. nucifera* var. *radicans*, respectively, separated by more than 17 mutation steps. Notably, this genetic distance exceeded the number of mutation steps observed between either variety and the outgroup *T. fargesii* Franch. (15 and 16 steps, respectively) (Figure 2; Table S1). For nuclear loci, there were three types of haplotype networks (Figure S1): (1) complete differentiation with predominantly private haplotypes for each variety at locus *T26*, *T82*, *T140*, and *T212*; (2) shared haplotypes between the two varieties at locus *T8*, *T173*, *T222*, and *T235*; (3) haplotypes shared among at least three of the four taxa (*T. nucifera* var. *nucifera*, *T. nucifera* var. *radicans*, *T. fargesii* and *T. jackii* Chun) at locus *T161*, *T147*, *T203*, *T249*, *T275*, and *T293*.

The genetic relationships constructed in the phylogenetic tree, estimated based on the partitioned nuclear data, revealed that all individuals were divided into two clades corresponding precisely to the two varieties with robust support (bootstrap value = 1.00) (Figure 2 and Figure S2). Consistent with the phylogenetic analyses, the genetic boundary between the two varieties was clearly identified using the Bayesian clustering algorithm based on 47 independently segregating sites. The most likely number of clusters was determined to be *K* = 2 based on both Ln*P*(*D*) and ∆*K* statistics. Each variety was found to contain less than 1% genetic admixture from the other (Figure 2).

The genetic differentiation (*F*_ST_) between *T. nucifera* var. *nucifera* and *T. nucifera* var. *radicans* exhibited an extremely high level (0.9619, *p* < 0.001) in chloroplast DNA. This level was comparable to that observed between other species pairs (Table 1). Similarly, high genetic differentiation between the two varieties and between other species pairs was also revealed at each nuclear locus (ranging from 0.0862 to 1.0000, *p* < 0.001) and across all loci (ranging from 0.6543 to 0.8089, *p* < 0.001) (Table 1 and Table S3).

### 2.3. Divergence and Demographic History

**Figure 3:**
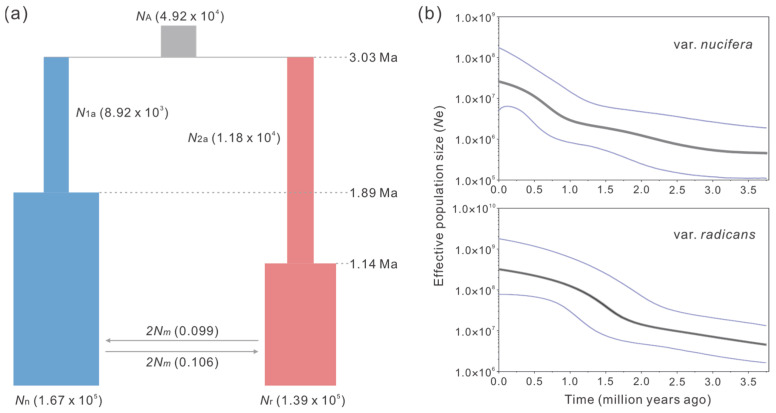
Demographic history of *T. nucifera* var. *nucifera* and *T. nucifera* var. *radicans* based on 14 nuclear loci. (**a**) Population divergence and demographic history of *T. nucifera* var. *nucifera* (*N*_n_) and *T. nucifera* var. *radicans* (*N*_r_) simulated by DIYABC, with exception of migration rates (*2Nm*) inferred using IM model. (**b**) Historical changes in effective population size (*N*_e_) depicted by Bayesian skyline plot in BEAST. The black and blue lines represent the median posterior and 95% highest posterior densities of effective population size over time, respectively.

Among the seven demographic scenarios evaluated, the ABC simulation identified Scenario 4 as the optimal model, exhibiting the highest posterior probability (81.83% estimated by direct approach and 73.13% by logistic approach) (Table S4). This scenario supports population expansion in both *T. nucifera* var. *nucifera* and *T. nucifera* var. *radicans* following their divergence from an ancestral population (Figure 3 and Figure S3). Posterior estimates for Scenario 4 indicate that the two varieties diverged by approximately 3.03 Ma (95% CI: 0.72–6.75 Ma). The effective population size of *T. nucifera* var. *nucifera* (1.67 × 10^5^, 95% CI: 0.17–3.66 × 10^5^) was comparable to that of *T. nucifera* var. *radicans* (1.39 × 10^5^, 95% CI: 0.10–3.57 × 10^5^). Population expansions were detected in both varieties, with the expansion in *T. nucifera* var. *nucifera* occurring earlier (1.89 Ma, 95% CI: 0.79–2.88 Ma) than that in *T. nucifera* var. *radicans* (1.14 Ma, 95% CI: 0.19–2.55 Ma) (Figure 3 and Figure S4; Table S5). A similar pattern of population expansion was also revealed in the Bayesian skyline plot analyses, with both varieties showing significant population growth since approximately 2.0 Ma (Figure 3).

The demographic parameters (except for gene flow) estimated from the IM model were generally lower than, but of similar magnitude to, those obtained from DIYABC (Table 2; Figure S5). The divergence time between the two varieties was estimated to be approximately 1.77 Ma (95% HPD: 1.26–8.37 Ma). The effective population size of *T. nucifera* var. *nucifera* (0.40 × 10^5^, 95% HPD: 0.26–0.69 × 10^5^) was significantly lower than that of *T. nucifera* var. *radicans* (1.34 × 10^5^, 95% HPD: 0.90–1.82 × 10^5^). Furthermore, the IM model analyses revealed low gene flow between the two varieties, with migration rates (*2Nm*) of 0.106 (95% HPD: 0.026–0.287) from *T. nucifera* var. *nucifera* to *T. nucifera* var. *radicans* and 0.099 (95% HPD: 0.028–0.310) in the opposite direction (Table 2; Figure 3).

### 2.4. Climatic Niche Differentiation

**Figure 4:**
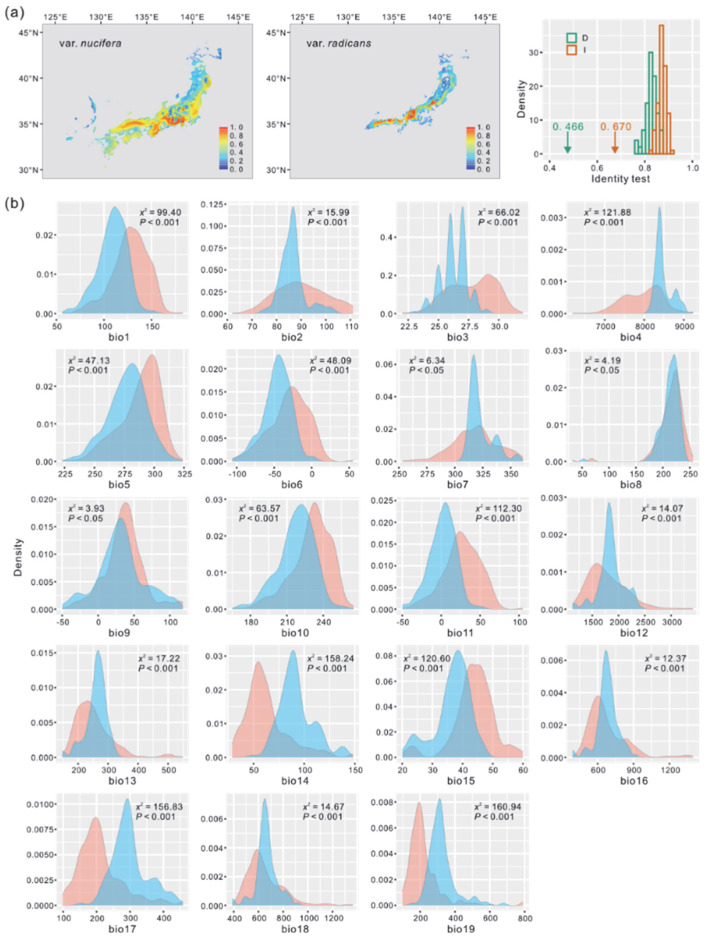
Climatic niche differentiation between *T. nucifera* var. *nucifera* and *T. nucifera* var. *radicans* in the Present. (**a**) Climatic niches modeled using MAXENT based on seven climatic vaiables with a correlation coefficient of *r* ≤ 0.70 and niche differentiation measured by identity tests (*I* and *D*) in ENMTools. (**b**) Kernel density plots of 19 climatic variables for *T. nucifera* var. *nucifera* (red curves) and *T. nucifera* var. *radicans* (blue curves). The differences in each ecological variable were assessed using nonparametric Kruskal–Wallis test with *χ*^2^ and *p* value.

Seven climatic variables (bio1, bio2, bio4, bio8, bio9, bio12, and bio15) with a correlation coefficient of *r* ≤ 0.70 were randomly selected for niche prediction of the two varieties using the MAXENT model. The predicted distributions of the two varieties remained relatively stable across the four periods (Present, MH, LGM, and LIG), with *T. nucifera* var. *nucifera* exhibiting a wider distribution than *T. nucifera* var. *radicans* (Figure 4 and Figures S6–S8). The niche models demonstrated excellent fit to the presence data, as indicated by high AUC values (>0.984).

The results from ENMTools showed that the observed values for *I* and *D* were significantly lower than those expected from pseudo-replicated datasets (*p* < 0.001) across all four periods (Figure 4 and Figures S6–S8), suggesting strong ecological differentiation between the two varieties. Furthermore, the Kruskal–Wallis test revealed significant differences (*p* < 0.05) in almost all climatic variables between the two varieties across the four periods, except for bio13, bio16, and bio18 during the MH; bio2, bio8, and bio9 during the LGM; and bio8 and bio16 during the LIG (*p* > 0.05) (Figure 4 and Figures S6–S8). These results further emphasize the role of climatic factors in maintaining the divergence between the two varieties.

## 3. Discussion

### 3.1. Recognition of Torreya nucifera var. nucifera and T. nucifera var. radicans as Two Separate Species Is Further Supported by Population Genetic Analyses

In almost all previous phylogenetic studies of *Torreya*, *T. nucifera* var. *nucifera* was conventionally designated as the proxy of *T. nucifera*, whereas *T. nucifera* var. *radicans* was usually excluded from sampling [25,26,27,28,29]. This sampling limitation likely arises from the taxonomic ambiguity of *T. nucifera* var. *radicans*. After the first publication of *T. nucifera* var. *radicans* [21], it was further elevated to species level, *T. fruticosa*, based on its form-shrubby morphology ([22]; Figure 1), or treated as a synonym of *T. nucifera* [23,24]. Also, Aizawa and Worth [19] supported treating *T. nucifera* var. *radicans* as a distinct species *T. fruticosa* based on phylogenetic analyses, although the data employed in the phylogenetic analyses were limited. In this study, we further reconciled this taxonomic controversy by population genetic analyses with integrating multiple robust lines, supporting that *Torreya nucifera* var. *nucifera* and *T. nucifera* var. *radicans* should be recognized as two separate species rather than varieties.

First, the network of chloroplast haplotypes revealed substantial genetic divergence between *T. nucifera* var. *nucifera* and *T. nucifera* var. *radicans* (17 mutation steps). Notably, this divergence exceeded that observed between either variety and the outgroup *T. fargesii* (15 and 16 steps, respectively) (Figure 2). A similar pattern was also found in several nuclear loci, such as *T82*, *T26*, *T140*, and *T212*, with exception of the loci that haplotypes were shared between two varieties which are likely a result of incomplete lineage sorting (Figure S2). Second, phylogenetic analysis based on nuclear loci showed reciprocal monophyly between *T. nucifera* var. *nucifera* and *T. nucifera* var. *radicans*, with both clades receiving robust support (bootstrap value = 1.00) (Figure 2 and Figure S3). Consistently, Bayesian clustering analyses revealed a clear genetic boundary between the two varieties, with extremely limited genetic admixture between them (<1%) (Figure 2). Third, the genetic differentiation (*F*_ST_) between the two varieties in chloroplast DNA displayed an extremely high level (0.9619, *p* < 0.001), which was comparable to that observed between other species pairs (Table 1). Although the genetic differentiation between the two varieties (0.6543, *p* < 0.001) was lower than that between either variety and the outgroups (*T. fargesii* and *T. jackii*) across all nuclear loci (Table 1 and Table S3), it was higher than that between the sister species *T. fargesii* and *T. yunnanensis* W. C. Cheng & L. K. Fu measured by using the same nuclear loci (0.5765; [30]). Fourth, the divergence time between the two varieties was dated back to the late Neogene or early Quaternary (3.03 Ma in DIYABC and 1.77 Ma in IMa2). These estimates are distinctly older than other speciation or incipient speciation events in the Japanese archipelago [6,8], such as that between *Phyllodoce caerulea* (L.) Bab. and *P. aleutica* (Spreng.) A. Heller (0.21–0.31 Ma) ([31]), and within *Cryptomeria japonica* (Thunb. ex L. f.) D. Don (0.32–0.85 Ma) [32]. Fifth, there are significant differences in the climatic niches and almost all climatic variables between the two varieties across the four time periods (Present, MH, LGM and LIG) (Figure 4 and Figures S6–S8). The observed climatic differences likely maintain the separation of the two varieties, potentially reducing gene flow between them, which was supported by the results from the IM model (Figure 3, Table 2). In summary, following the taxonomic treatment of Aizawa and Worth [19] based on phylogenetic analyses, we further support that the two varieties should be recognized as two separate species based on population genetic analyses, and the previous nomenclature, *T. fruticose* [22], should be restored to replace *T. nucifera* var. *radicans*.

### 3.2. Allopatric Origin Might Be Applicable for T. nucifera var. nucifera and T. nucifera var. radicans

The mode of in situ speciation or population differentiation has frequently been uncovered in phylogeographic studies of various plant groups in the Japanese archipelago (e.g., [7,8,33,34,35,36]). The climate oscillations during the late Quaternary, especially the last glacial–interglacial cycles, profoundly influenced in situ diversification by range fragmentation, vicariance, and population isolation in many plant species [6,14]. As a result, inter- or intra-specific divergence occurred relatively recently, typically dating back as far as the late Quaternary, as confirmed by demographic inferences for many plant species in the Japanese archipelago [6,8]. However, our study found that *T. nucifera* var. *nucifera* and *T. nucifera* var. *radicans* have a rather high genetic differentiation (*F*_ST_) between them (0.9619 in chloroplast DNA and 0.6543 across all nuclear loci) (Table 1; Figure 2), and that they diverged in the early Quaternary (1.77 Ma) or late Neogene (3.03 Ma), estimated through two prevalent coalescent simulations with different algorithms, the IM model and the DIYABC, respectively (Figure 3; Table 2). Although the estimated time of the two approaches is different, their confidence intervals almost overlap (1.26–8.37 Ma in the IM model and 0.72–6.75 Ma in the DIYABC); this difference has also been observed in other studies on population divergence history (e.g., [30,37]). To our knowledge, these estimates are distinctly older than those of in situ speciation or population differentiation events in the Japanese archipelago [6,8], like the divergence between *P. caerulea* and *P. aleutica* (0.21–0.31 Ma) [31] and within *C. japonica* (0.32–0.85 Ma) [32]. Therefore, in situ speciation might not be a good explanation for the origin of the two varieties.

An alternative hypothesis posits that the modern flora in East Asia originated allopatrically at the high latitudes of the Northern Hemisphere and then became restricted to current regions (refugia) at the lower latitudes as a result of global cooling over the last 15 million years [38]. The *Torreya* species is one of the representative taxa of the floras. Although there is a lack of convincing fossil records for *Torreya*, its historical biogeography has been well inferred based on the divergence times indicated by molecular data, approximately 17 Ma (the early Miocene) estimated for the basal split of extant species in the genus [39,40]. However, the divergence time between *T. nucifera* var. *nucifera* and *T. nucifera* var. *radicans*, which was inferred to be 1.77 Ma in the IM model or 3.03 Ma in the DIYABC approach in the present study (Figure 3; Table 2), is much lower than that of the basal split of extant species in *Torreya* (approximately 17 Ma). Also, the divergence time is far below that between *F. longipetiolata* Seemen and *F. lucida* Rehder & E. H. Wilson, which diverged allopatrically in the Late Miocene (approximately 8.5 Ma) at the high latitudes of the Northern Hemisphere [37]. Therefore, it is improbable that the two varieties speciated allopatrically at the high latitudes of the Northern Hemisphere.

Furthermore, the most plausible speciation scenario for *T. nucifera* var. *nucifera* and *T. nucifera* var. *radicans* is that they originated allopatrically due to the range fragmentation of their ancestor in East Asia. Subsequently, range shifts during the late Neogene and the early Quaternary led to their assembly in the Japanese archipelago. The timing of the two varieties’ divergence was inferred to have occurred at the early Quaternary or late Neogene in the present study (Figure 3; Table 2). During these periods, the continental shelf across the East China Sea was considered to have been exposed due to sea-level fluctuations [6,14]. Consequently, this exposure created conditions for the allopatric origin of *T. nucifera* var. *radicans*, or allopatric hybridization between *T. grandis* and *T. nucifera* var. *nucifera*. In addition, this scenario was supported by the phylogenetic inference of *Torreya* based on chloroplast DNA fragments and nuclear ITS sequences [19]. Specifically, the variety *T. nucifera* var. *radicans* probably descended from a most recent common ancestor with a closely related species *T. grandis* endemic in China or originated from allopatric hybridization between *T. grandis* and *T. nucifera* var. *nucifera*. However, the scenario regarding allopatric origin of *T. nucifera* var. *nucifera* and *T. nucifera* var. *radicans* still demands more evidence, especially from demographic history inferred based on population sampling comprising all Asian extant species of *Torreya*.

### 3.3. Climatic Heterogeneity Sustains the Species Boundary Between T. nucifera var. nucifera and T. nucifera var. radicans

Climate oscillations during the Quaternary have been speculated as a main cause of maintaining and promoting plant diversification in the Japanese archipelago [6,8]. The present distributions of *T. nucifera* var. *nucifera* and *T. nucifera* var. *radicans* are mainly on the Pacific Ocean side and the Japan Sea side of the Japanese archipelago, respectively (Figure 1). This distribution pattern is primarily determined by climatic differences between the two sides due to the Asian monsoon and the Tsushima Current, namely a dry climate in winter on the Pacific Ocean side and heavy snowfall during winter on the Japan Sea side [7,15,19]. In this study, significant differences in the climatic niches and almost all climatic variables were detected between *T. nucifera* var. *nucifera* and *T. nucifera* var. *radicans* across the four time periods (Present, MH, LGM and LIG) (Figure 4 and Figures S6–S8). This result suggests that climatically based divergent selection has played a crucial role in maintaining the separation of the two varieties, potentially by limiting gene flow between them. Although extremely weak gene flow between the two varieties was detected in the IM model (Figure 3; Table 2), this might be due to the increase in the population sizes of both varieties since the early Quaternary (approximately 2.0 Ma) inferred in the Extended Bayesian skyline plot (EBSP) analysis (Figure 3). Similarly, the patterns of population genetic divergence across the Pacific Ocean side and the Japan Sea side driven by the Asian monsoon and the Tsushima Current were also revealed in other plant taxa, such as *F. crenata* [33] and *C. japonica* [41].

## 4. Materials and Methods

### 4.1. Sampling and DNA Sequencing

Six populations of *T. nucifera*, comprising 31 individuals from three populations of *T. nucifera* var. *nucifera* and 52 individuals from three populations of *T. nucifera* var. *radicans*, were collected (Figure 1; Table S1). Leaves were dried in silica gel immediately after collection and subsequently used for DNA extraction. Total genomic DNA was extracted from approximately 20 mg of dried leaves using a modified CTAB method. Additionally, nuclear loci datasets of *T. jackii* and *T. fargesii* from our previous studies [30,42] were used as outgroups.

Three chloroplast (cp) DNA regions (*rpl*16, *rpo*B-*trn*C and *trn*L-*trn*F) and 14 low-copy nuclear genes (*T8*, *T26*, *T82*, *T140*, *T147*, *T161*, *T173*, *T203*, *T212*, *T222*, *T235*, *T249*, *T275*, and *T293*), developed from the transcriptome sequences of *T. grandis* [43], were amplified using polymerase chain reaction (PCR) following protocols from our previous studies [26,30]. The PCR products were purified and sequenced by Sangon Biotech (Shanghai, China). Sequences of the same locus were aligned and verified using MEGA X [44].

### 4.2. Genetic Diversity Analyses and Neutrality Tests

Because the chloroplast genome is inherited uniparentally in gymnosperms [45], the three cpDNA regions were concatenated into a single matrix to estimate population genetic parameters. The number of haplotypes (*N*_h_), haplotype diversity (*H*_d_), and nucleotide diversity (*π*) were calculated using DnaSP 5.10 [46]. Tajima’s *D* [47] and Fu’s *F*_S_ [48] were computed using Arlequin 3.5 [49].

Nuclear sequences were initially assigned to coding and non-coding regions by aligning them with their corresponding transcriptome sequences [43]. For each nuclear locus, population genetic parameters, the number of segregating sites (*S*), the number of haplotypes (*N*_h_), haplotype diversity (*H*_d_), nucleotide diversity (*π*), the Watterson parameter (*θ*_w_), and the minimum number of recombination events (*R*_m_) were estimated after sequence phasing using the PHASE algorithm in DnaSP 5.10 [46] with default parameters.

The expectation of neutral evolution was assessed for each locus using Tajima’s *D*, Fu and Li’s D* and F* tests [50] in DnaSP 5.10 [46]. Under neutrality, these parameters are expected to approach zero, which was evaluated by comparing the observed values of the summary statistics with their expected distributions.

### 4.3. Analyses of Population Genetic Structure

The relationships among haplotypes for each locus were constructed using the median-joining network method in Network 5.0 (http://www.fluxus-engineering.com). Wright’s fixation index (*F*_ST_, [51]) among species was calculated for each nuclear locus using AMOVA in Arlequin 3.5 [49]. The significance of *F*_ST_ values was assessed based on 10,000 permutations. Additionally, phylogenetic relationships among species were inferred using BEAST 2.7.1 [52] based on partitioned nuclear data, employing a relaxed-clock model and a Yule speciation process. Two independent Markov chain Monte Carlo (MCMC) runs were conducted, each with 20,000,000 generations, sampling every 100,000 generations and discarding the first 20% as burn-in. The appropriate substitution model for sequence evolution in the phylogenetic analyses was selected using jModelTest 2.1.10 [53].

Population structure was analyzed using the admixture model implemented in the Bayesian assignment algorithm in STRUCTURE 2.3.4 [54], based on the dataset of 14 nuclear loci. Segregating sites exhibiting significant linkage disequilibrium after Bonferroni correction in DnaSP 5.10 [46] were excluded from the analysis. The number of clusters (*K*), varying from 1 to 6, was tested with 20 independent runs performed for each *K*. The burn-in was set to 20,000, followed by 200,000 MCMC runs. The most likely number of clusters was determined using Ln*P*(*D*) [55] and Δ*K* statistics [56]. The resulting population clusters were visualized using the program DISTRUCT 1.1 [57].

### 4.4. Inferences of Divergence and Demographic History

The divergence and demographic history of the two varieties were investigated using an Approximate Bayesian Computation (ABC) approach and the Isolation-with-Migration (IM) model based on nuclear data. Based on analyses of genetic diversity, population structure, and neutrality tests, seven possible scenarios of population divergence and demography (Figure S1) were formulated and modeled using DIYABC 2.1.0 [58]. These scenarios included: (i) *T. nucifera* var. *nucifera* and *T. nucifera* var. *radicans* diverging from an ancestral population at time *t*_2_, followed by a population expansion event in (ii) *T. nucifera* var. *nucifera* at time *t*_0_, or in (iii) *T. nucifera* var. *radicans* at time *t*_1_, or in (iv) both *T. nucifera* var. *nucifera* at time *t*_0_ and *T. nucifera* var. *radicans* at time *t*_1_. Alternatively, the two varieties diverged from an ancestral population at time *t*_2_, followed by a population contraction event in (v) *T. nucifera* var. *nucifera* at time *t*_0_, or in (vi) *T. nucifera* var. *radicans* at time *t*_1_, or in (vii) both *T. nucifera* var. *nucifera* at time *t*_0_ and *T. nucifera* var. *radicans* at time *t*_1_. The priors for all parameters were set with a uniform distribution (Table S6) after repeated attemps starting from a larger range of initial values for each parameter. All one-sample and two-sample summary statistics were selected to compare the observed and simulated datasets. To ensure statistical robustness, at least 1,000,000 simulated datasets were generated for each scenario. The 1% of the simulated datasets closest to the observed data were used to estimate the relative posterior probability through logistic regression and posterior parameter distributions with 95% confidence intervals (CIs). A generation time of 25 years, previously applied to *Taxus wallichiana* Zucc. in the same family (Taxaceae) [59], was adopted to scale the population divergence and demographic history of the two varieties in the present study.

The population divergence was further estimated using the IM model. After extracting the longest non-recombining region of each locus using DnaSP 5.10 [46], the demographic parameters, including migration rate (*m*) and divergence time (*t*) between *T. nucifera* var. *nucifera* and *T. nucifera* var. *radicans* and their effective population size (*θ*) were simulated and estimated through the MCMC approach implemented in the IMa2 software [60]. The simulations were run with a burn-in of 2,000,000 steps, followed by 100,000 retained steps under the HKY mutation model. The demographic parameters obtained from the IM model were scaled by a mean mutation rate (2.23 × 10^−10^ per site per year [30]).

The temporal changes in the effective population sizes of *T. nucifera* var. *nucifera* and *T. nucifera* var. *radicans* were inferred using the Extended Bayesian skyline plot (EBSP) analysis in BEAST 2.7.1 [52] based on the partitioned nuclear data. The MCMC chains were run for 20,000,000 generations, with sampling every 100,000 generations and a burn-in of 20%. The substitution model for sequence evolution was selected using jModelTest 2.1.10 [53]. Tracer v.1.7.1 [61] was used to verify that the effective sample size (ESS) was not less than 200 and to generate the produce Skyline plots.

### 4.5. Niche Modeling and Ecological Divergence

To estimate the effect of ecological factors on population genetic divergence, the climatic niches of the two Japanese *Torreya* taxa were simulated and statistically compared. A total of 310 occurrence records of *T. nucifera* var. *nucifera* and 136 records of *T. nucifera* var. *radicans* were retrieved from the Global Biodiversity Information Facility (GBIF, https://www.gbif.org) (Figure 1) and used for niche modeling and ecological divergence analyses after excluding mistake, imprecise and duplicate records. Nineteen bioclimatic variables for four periods, the Present, the Mid-Holocene (MH), the Last Glacial Maximum (LGM), and the Last Interglacial (LIG) under MIROC model, were downloaded from WorldClim database with a spatial resolution of 2.5 min for each environmental layer (https://www.worldclim.org [62]). To minimize overfitting of the niche models, ecological variables with pairwise Pearson correlation coefficients of *r* ≤ 0.70 were retained for subsequent analyses.

The climate niches of the two varieties were modeled using MAXENT 3.4.3 [63] with default parameters. The species records were split into 80% for model training and 20% for model testing, with ten replicates performed for each run. Model accuracy was evaluated using the area under the ROC curve (AUC). An AUC value above 0.9 was considered as a good model performance [64]. Additionally, niche differences among the two varieties were quantified using Schoener’s *D* (*D*) and a standardized version of Hellinger distance (*I*) in ENMTools 1.4.4 [65,66]. An identity test was conducted to compare niche distributions, based on 100 pseudo-replicates generated through random sampling of pooled occurrence records from both varieties. Furthermore, differences in each of the 19 bioclimatic variables between the two varieties were assessed using the nonparametric Kruskal–Wallis test and visualized using kernel density plots drew by ggplot2 package in R 3.5.2.

## 5. Conclusions

In this research, the influences of climatic oscillations during the Quaternary on species diversification in the Japanese archipelago were explored by inferring the divergence history of two Japanese *Torreya* taxa using population genetic analyses. The results showed that *T. nucifera* var. *nucifera* and *T. nucifera* var. *radicans* have extremely high genetic differentiation, a distinct genetic boundary, and significant climatic niche differentiation. These findings further support the two Japanese *Torreya* taxa as two separate species following the taxonomic treatment of Aizawa and Worth [19] based on phylogenetic analyses. Furthermore, in contrast to the numerous cases of in situ speciation uncovered in the Japanese archipelago, a relatively ancient divergence time between the two *Torreya* taxa was estimated through two coalescent simulations with different algorithms (IM model and DIYABC). This finding suggests that they likely speciated allopatrically due to the sea-level fluctuations associated with climatic oscillations during the Quaternary. Significant ecological differences have played a crucial role in maintaining species boundary after speciation of the two *Torreya* taxa. However, more details for allopatric origin of the two *Torreya* taxa is still required, especially from inferring demographic history based on population sampling comprising all Asian extant species of *Torreya*. Overall, our findings demonstrate that vicariant allopatric speciation may be a common mode of speciation that occurred under climatic oscillations during the Quaternary and potentially contributed to the present-day species diversification in the Japanese archipelago.

## Figures and Tables

**Table 1:** Genetic differentiation (*F*_ST_) between each pair of species of *Torreya nucifera* var. *nucifera*, *T. nucifera* var. *radicans*, and outgroups (*T. fargesii* and *T. jackii*), based on AMOVA for chloroplast DNA (lower left) and 14 nuclear loci (upper right).

	var. *nucifera*	var. *radicans*	*T. fargesii*	*T. jackii*
var. *nucifera*		0.6543 *	0.7288 *	0.8089 *
var. *radicans*	0.9619 *		0.7904 *	0.7944 *
*T. fargesii*	0.9760 *	0.9510 *		0.8033 *
*T. jackii*	0.9866 *	0.9648 *	0.9764 *	

*, significant level at *p* < 0.001.

**Table 2:** Maximum likelihood estimates (MLEs) and 95% highest posterior density (HPD) of demographic parameters for *T. nucifera* var. *nucifera* (n) and *T. nucifera* var. *radicans* (r) inferred using IM model.

	*θ*_n_	*θ*_r_	*θ*_A_	*m*_n>r_	*m*_r>n_	*t*	*N*_n_	*N*_r_	*N*_A_	*2Nm* (n)	*2Nm* (r)	*T* (Years)
MLE	0.152	0.511	0.862	1.307	0.415	1.692	39,668	133,667	225,702	0.106	0.099	1,772,099
HPD95Lo	0.100	0.345	0.070	0.562	0.153	1.204	26,157	90,202	18,328	0.026	0.028	1,260,997
HPD95Hi	0.263	0.695	3.818	2.354	0.827	7.996	68,889	181,844	999,686	0.287	0.310	8,374,529

*θ* and *N*, effective population size; *m* and *2Nm*, population migration rate; *t* and *T*, divergent time. *θ*, *m* and *t* were scaled by the mutation rate, while *N*, *2Nm*, and *T* were scaled by individuals or years.

## Data Availability

The chloroplast and nuclear DNA sequences obtained in the present study is available at figshare repository (https://doi.org/10.6084/m9.figshare.28735670.v1).

## Supplementary Materials

The following supporting information can be downloaded at: https://www.mdpi.com/article/10.3390/plants14101537/s1, Table S1: Sampling information, the distribution of chloroplast haplotypes, and chloroplast genetic diversity of *Torreya nucifera* var. *nucifera* and *T. nucifera* var. *radicans*; Table S2: Nucleotide diversity, haplotype diversity and neutrality test across 14 nuclear loci in *T. nucifera* var. *nucifera* and *T. nucifera* var. *radicans*; Table S3: Genetic differentiation (*F*_ST_) among each two species of *T. nucifera* var. *nucifera* (nuc), *T. nucifera* var. *radicans* (rad), *T. fargesii* (far) and *T. jackii* (jac) for each nuclear locus and across all loci; Table S4: Posterior probabilities for seven scenarios formulated in Figure S1 modeled by DIYABC based on 14 nuclear loci; Table S5: Posterior estimates of demographic parameters for the best scenario (scenario 4) simulated by DIYABC based on 14 nuclear loci; Table S6: The prior distribution of parameters for all scenarios in the simulations of DIYABC; Figure S1: Haplotype genealogies of 14 nuclear loci for *T. nucifera* var. *nucifera* (blue circles) and *T. nucifera* var. *radicans* (red circles). Haplotypes of *T. fargesii* (green circles) and *T. jackii* (yellow circles) were used as the outgroups; Figure S2: Phylogenetic relationships among *T. nucifera* var. *nucifera*, *T. nucifera* var. *radicans*, *T. fargesii* and *T. jackii* were inferred using BEAST based on the partitioned nuclear datasets (14 nuclear loci); Figure S3: Seven possible scenarios for population divergence and demography of *T. nucifera* var. *nucifera* and *T. nucifera* var. *radicans* simulated in DIYABC; Figure S4: Prior and posterior distributions (Scenario 4 in Figure 1) of demographic parameters for *T. nucifera* var. *nucifera* (n) and *T. nucifera* var. *radicans* (r) estimated using DIYABC; Figure S5: Posterior probability distributions of effective population size (*θ*), migration rate (*m*) and divergence time (*t*) between *T. nucifera* var. *nucifera* (n) and *T. nucifera* var. *radicans* (r) estimated using IM model based on 14 nuclear loci; Figure S6: Climatic niche differentiation between *T. nucifera* var. *nucifera* and *T. nucifera* var. *radicans* in the Mid-Holocene (MH); Figure S7: Climatic niche differentiation between *T. nucifera* var. *nucifera* and *T. nucifera* var. *radicans* in the Last Glacial Maximum (LGM); Figure S8: Climatic niche differentiation between *T. nucifera* var. *nucifera* and *T. nucifera* var. *radicans* in the Last Interglacial (LIG).
