# Supplementary material for: The Divergence History of Two Japanese Torreya Taxa (Taxaceae): Implications for Species Diversification in the Japanese Archipelago
Source: Plants (Basel). 2025 May 20;14(10):1537. doi: 10.3390/plants14101537 (PMC12114668; doi:10.3390/plants14101537)
Supplement: Supplementary file 1 [file plants-14-01537-s001.zip › plants-3622544-supplementary.pdf]

Table S1. Sampling information, the distribution of chloroplast haplotypes, and chloroplast genetic diversity of *Torreya nucifera* var. *nucifera* and *T. nucifera* var. *radicans*.

Population/ Location	Latitude (°N)	Longitude (°E)	N_s	H1	H2	H3	H4	H5	H6	H7	H8	H_d	π
var. *nucifera*													
1. Tokyo	35.6252	139.2547	8	8	0	0	0	0	0	0	0	0	0
2. Kanagawa	35.4614	139.2131	17	14	1	1	1	0	0	0	0	0.3309	0.00014
3. Nagano	35.6750	137.6161	6	5	0	0	0	1	0	0	0	0.3333	0.00013
Total			31	27	1	1	1	1	0	0	0	0.5935	0.00008
var. *radicans*													
1. Gifu	35.6519	136.6764	12	0	0	0	0	0	12	0	0	0	0
2. Fukui	35.5060	135.4784	19	0	0	0	0	0	15	2	2	0.3743	0.00016
3. Hyogo	35.2156	134.5000	21	0	0	0	0	0	16	5	0	0.3810	0.00015
Total			52	0	0	0	0	0	43	7	2	0.3024	0.00013

N_s, numbers of individual; H_d, haplotype diversity; π, nucleotide diversity.

Table S2. Nucleotide diversity, haplotype diversity and neutrality test across 14 nuclear loci in *T. nucifera* var. *nucifera* and *T. nucifera* var. *radicans*.

Variety	Locus	Total					Haplotype diversity		Neutrality tests		
		N	L	S	π	θ_w	N_h	H_d	D	D^*	F^*
var. *nucifera*	T8	31	787	3	0.00104	0.00081	4	0.644	0.55626	0.87014	0.90336
	T26	31	290	1	0.00032	0.00073	2	0.094	-0.71361	0.52555	0.18954
	T82	31	287	0	0.00000	0.00000	1	0.000	-	0.00000	0.00000
	T140	31	333	1	0.00019	0.00064	2	0.063	-0.89387	0.52555	0.12850
	T147	31	680	15	0.00835	0.00470	4	0.561	**2.30491***	0.60639	1.41989
	T161	31	388	1	0.00016	0.00055	2	0.063	-0.89387	0.52555	0.12850
	T173	31	417	0	0.00000	0.00000	1	0.000	-	0.00000	0.00000
	T203	31	407	11	0.00566	0.00576	8	0.786	-0.04452	0.83320	0.63393
	T212	31	448	1	0.00021	0.00048	2	0.094	-0.71361	0.52555	0.18954
	T222	31	407	2	0.00195	0.00105	3	0.650	1.46572	0.72630	1.10292
	T235	31	379	2	0.00033	0.00112	2	0.063	-1.19107	0.72630	0.17635
	T249	31	420	5	0.00410	0.00253	7	0.803	1.42995	1.07782	1.39508
	T275	31	405	1	0.00008	0.00053	2	0.032	-1.08044	-1.90277	-1.92699
	T293	31	427	1	0.00015	0.00050	2	0.063	-0.89387	0.52555	0.12850
	Average	31	434	3.14	0.00161	0.00139	3.00	0.280	-0.05567	0.39751	0.31922
var. *radicans*	T8	52	788	11	0.00153	0.00268	11	0.713	-1.10723	0.08608	-0.39955
	T26	52	290	2	0.00180	0.00132	3	0.514	0.55711	-1.08040	-0.67206
	T82	52	287	0	0.00000	0.00000	1	0.000	-	0.00000	0.00000
	T140	52	333	2	0.00089	0.00115	3	0.286	-0.34957	0.68236	0.42530
	T147	52	680	24	0.00783	0.00705	15	0.875	0.33059	**1.85625****	1.51900
	T161	52	388	4	0.00376	0.00198	5	0.671	1.77418	0.93733	1.42031
	T173	52	417	2	0.00023	0.00092	3	0.093	-1.15220	-1.08040	-1.28999
	T203	52	407	10	0.00813	0.00471	7	0.757	1.84835	1.37038	**1.82662***
	T212	52	445	2	0.00044	0.00086	3	0.194	-0.73900	0.68236	0.28452
	T222	52	407	5	0.00177	0.00235	5	0.537	-0.52519	-0.03444	-0.23053

T235	52	379	2	0.00093	0.00101	3	0.344	-0.12587	0.68236	0.50616
T249	52	420	4	0.00389	0.00183	5	0.716	**2.22692***	0.93733	1.59207
T275	52	405	3	0.00059	0.00142	4	0.229	-1.04420	0.82348	0.27359
T293	52	427	1	0.00111	0.00045	2	0.473	1.67129	0.48990	0.98564
Average	52	434	5.14	0.00235	0.00198	5.00	0.457	0.25886	0.45376	0.44579

N, number of individuals; *L*, sequence length of each locus; *S*, number of segregating sites; π, nucleotide diversity; θ, Watterson's parameter; N_h, number of haplotypes; H_d, Nei's haplotype diversity; R_m, minimum number of recombinant events; *D*, Tajima's *D* statistic; *D** and *F**, Fu and Li's *D** and Fu and Li's *F**; -, failed to be computed for lack of enough variation; *and**, significant level at $P < 0.05$ and $P < 0.01$, respectively.

Table S3. Genetic differentiation (F_{ST}) among each two species of *T. nucifera* var. *nucifera* (nuc), *T. nucifera* var. *radicans* (rad), *T. fargesii* (far) and *T. jackii* (jac) for each nuclear locus and across all loci.

	nuc-rad	nuc-far	rad-far	nuc-jac	rad-jac	far-jac
T8	0.51895*	0.90157*	0.89259*	0.95642*	0.94133*	0.94543*
T26	0.76894*	0.75711*	0.80503*	0.96895*	0.91944*	0.92022*
T82	1.00000*	0.95692*	0.95893*	1.00000*	1.00000*	0.87971*
T140	0.90595*	0.98413*	0.95529*	0.95363*	0.93777*	0.99448*
T147	0.24626*	0.49168*	0.51067*	0.39809*	0.54873*	0.49393*
T161	0.36361*	0.84897*	0.78076*	0.48558*	0.50374*	0.60930*
T173	0.94332*	0.92472*	0.84886*	0.99457*	0.96534*	0.84699*
T203	0.21735*	0.73157*	0.64954*	0.67492*	0.50385*	0.74093*
T212	0.97422*	0.90348*	0.95496*	0.98427*	0.98307*	0.91041*
T222	0.72703*	0.92915*	0.88903*	0.77017*	0.66790*	0.81246*
T235	0.78151*	0.96350*	0.93945*	0.97895*	0.94748*	0.98765*
T249	0.08617*	0.36338*	0.39142*	0.35493*	0.40498*	0.43948*
T275	0.85765*	0.06492*	0.81506*	0.99456*	0.96359*	0.96529*
T293	0.76981*	0.38200*	0.67389*	0.80888*	0.83416*	0.69941*
Average	0.65434*	0.72879*	0.79039*	0.80885*	0.79438*	0.80326*

*, significant level at $P < 0.001$.

Table S4. Posterior probabilities for seven scenarios formulated in Figure S1 modeled by DIYABC based on 14 nuclear loci.

Method	Scenario	Posterior probability	95% Credibility interval
Direct approach	Scenario 1	0.00719	0.00000–0.05532
	Scenario 2	0.11866	0.00000–0.31830
	Scenario 3	0.05016	0.00000–0.18075
	**Scenario 4**	**0.81828**	**0.58187–1.00000**
	Scenario 5	0.00057	0.00000–0.00994
	Scenario 6	0.00511	0.00000–0.04651
	Scenario 7	0.00000	0.00000–0.00000
Logistic approach	Scenario 1	0.00465	0.00000–0.02776
	Scenario 2	0.16921	0.08166–0.25892
	Scenario 3	0.08710	0.04397–0.13904
	**Scenario 4**	**0.73131**	**0.63061–0.83121**
	Scenario 5	0.00003	0.00000–0.01169
	Scenario 6	0.00771	0.00000–0.02705
	Scenario 7	0.00000	0.00000–0.00000

The scenario with the highest posterior probability was shown in bold font.

Table S5. Posterior estimates of demographic parameters for the best scenario (scenario 4) simulated by DIYABC based on 14 nuclear loci.

Parameter	mean	median	mode	95% CI
N_n	1.67×10^5	1.55×10^5	1.94×10^4	$0.17–3.66\times10^5$
N_r	1.39×10^5	1.08×10^5	0.94×10^4	$0.10–3.57\times10^5$
N_{1a}	0.89×10^4	0.88×10^4	0.81×10^4	$0.35–1.46\times10^4$
N_{2a}	1.18×10^4	1.19×10^4	1.18×10^4	$0.51–1.79\times10^4$
N_A	4.92×10^4	4.88×10^4	4.44×10^4	$2.74–7.36\times10^4$
T_0 (years)	1.89×10^6	1.93×10^6	2.15×10^6	$0.79–2.88\times10^6$
T_1 (years)	1.14×10^6	9.95×10^5	5.75×10^5	$0.19–2.55\times10^6$
T_2 (years)	3.03×10^6	2.58×10^6	1.27×10^6	$0.72–6.75\times10^6$

Table S6. The prior distribution of parameters for all scenarios in the simulations of DIYABC.

Parameter	Distribution	Minimum	Maximum
Effective population size			
N_1	Uniform	10	10000000
N_2	Uniform	10	10000000
N_{1a}	Uniform	10	500000
N_{2a}		10	500000
N_{1b}		10	2000000
N_{2b}	Uniform	10	2000000
N_a	Uniform	600000	2000000
Time of events (in generations backward in time)			
t_0	Uniform	10	120000
t_1	Uniform	10	120000
t_2	Uniform	10	300000
Mean mutation rate			
μ	Uniform	1×10^{-9}	1×10^{-7}

N_1 and N_2, current population sizes of *T. nucifera* var. *nucifera* and *T. nucifera* var. *radicans*; N_a, ancestral population size; N_{1a}, N_{2a}, N_{1b} and N_{2b}, population sizes between ancestral population and current population of *T. nucifera* var. *nucifera* and *T. nucifera* var. *radicans*, and $N_{1a} < N_1$, $N_{2a} < N_2$, $N_{1b} > N_1$ and $N_{2b} > N_2$; t_0, t_1 and t_2, times of population changes, and $t_2 > t_1$ and $t_2 > t_0$.

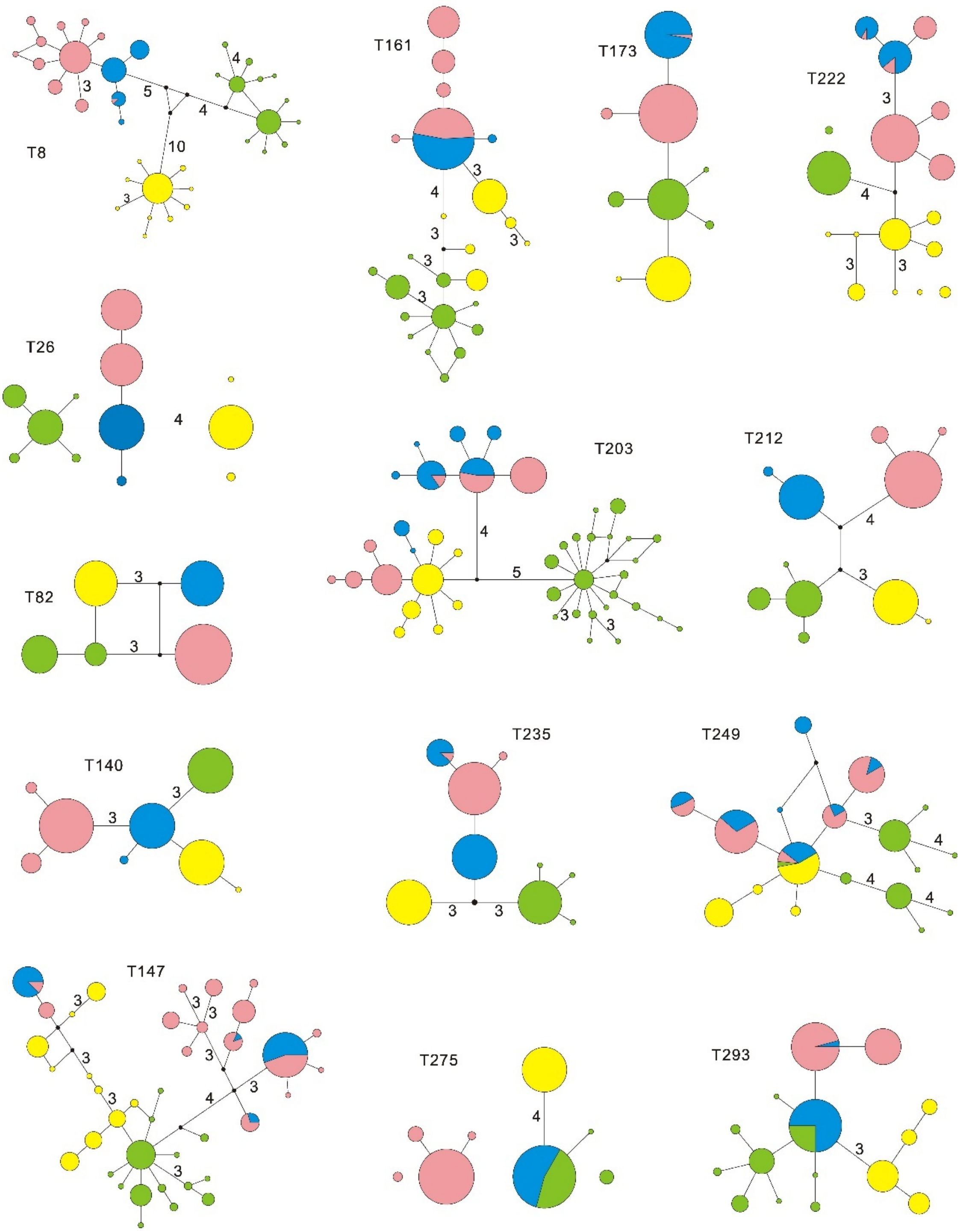

Figure S1. Haplotype genealogies of 14 nuclear loci for *T. nucifera* var. *nucifera* (blue circles) and *T. nucifera* var. *radicans* (red circles). Haplotypes of *T. fargesii* (green circles) and *T. jackii* (yellow circles) were used as the outgroups. The sizes of circles are proportional to the haplotype frequencies, and the mutation steps more than one are labeled with the corresponding numbers on each branch.

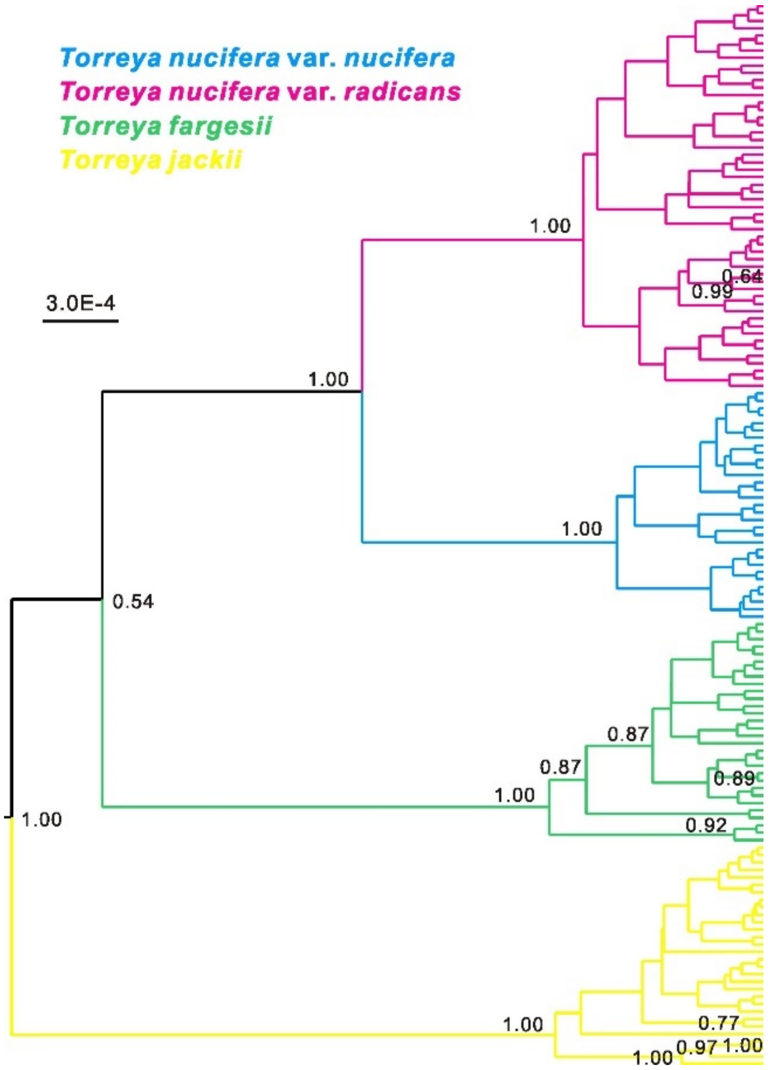

Figure S2. Phylogenetic relationships among *T. nucifera* var. *nucifera*, *T. nucifera* var. *radicans*, *T. fargesii* and *T. jackii* were inferred using BEAST based on the partitioned nuclear datasets (14 nuclear loci). Posterior values greater than 0.7 are labeled on the clades.

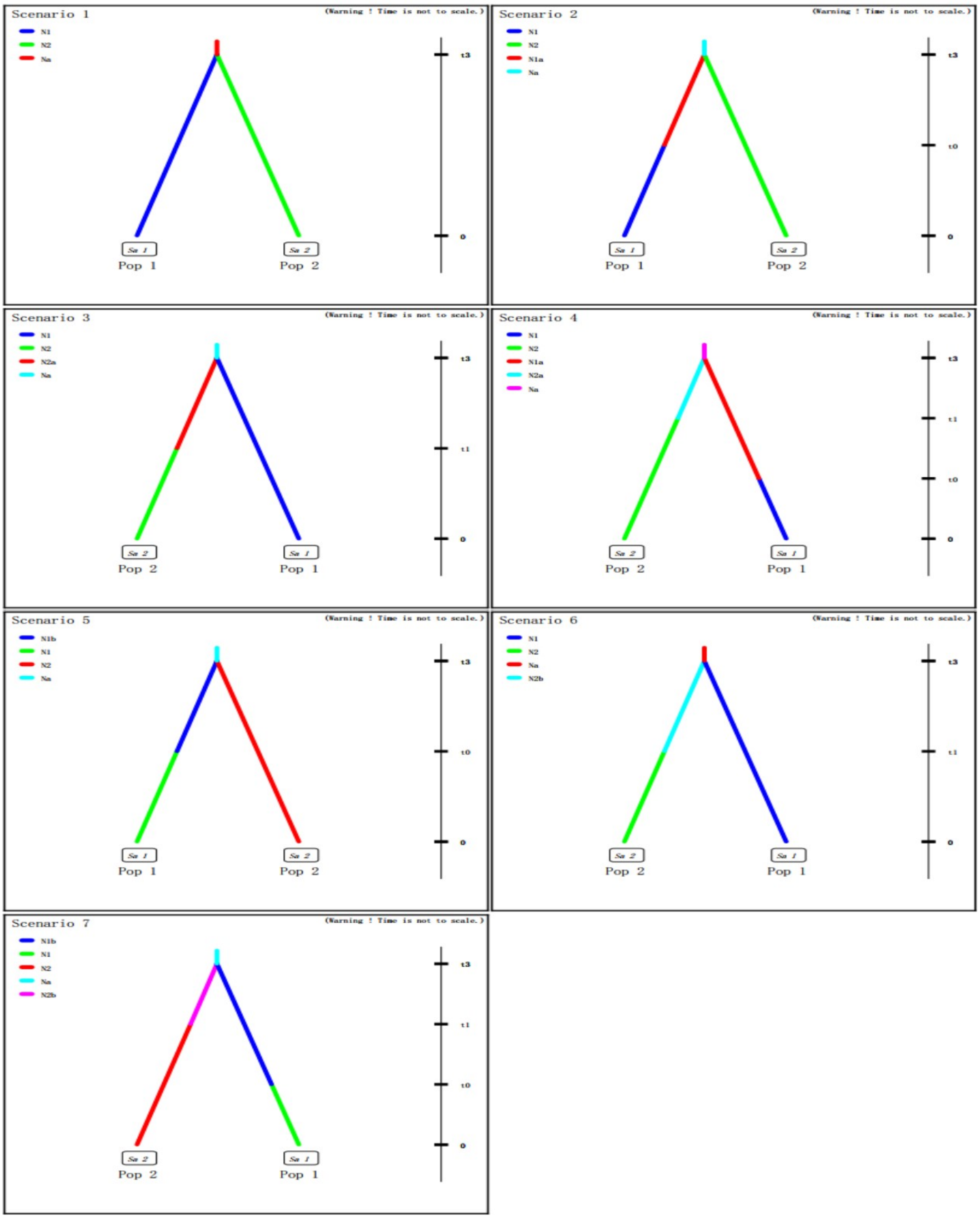

Figure S3. Seven possible scenarios for population divergence and demography of *T. nucifera* var. *nucifera* and *T. nucifera* var. *radicans* simulated in DIYABC. N_1 and N_2 separately represent current population sizes of *T. nucifera* var. *nucifera* and *T. nucifera* var. *radicans*, and N_a represents their ancestral population size. N_{1a}, N_{1b}, N_{2a} and N_{2b} represent population sizes between the ancestral population and the current populations. t_0, t_1 and t_2 represent times of population changes.

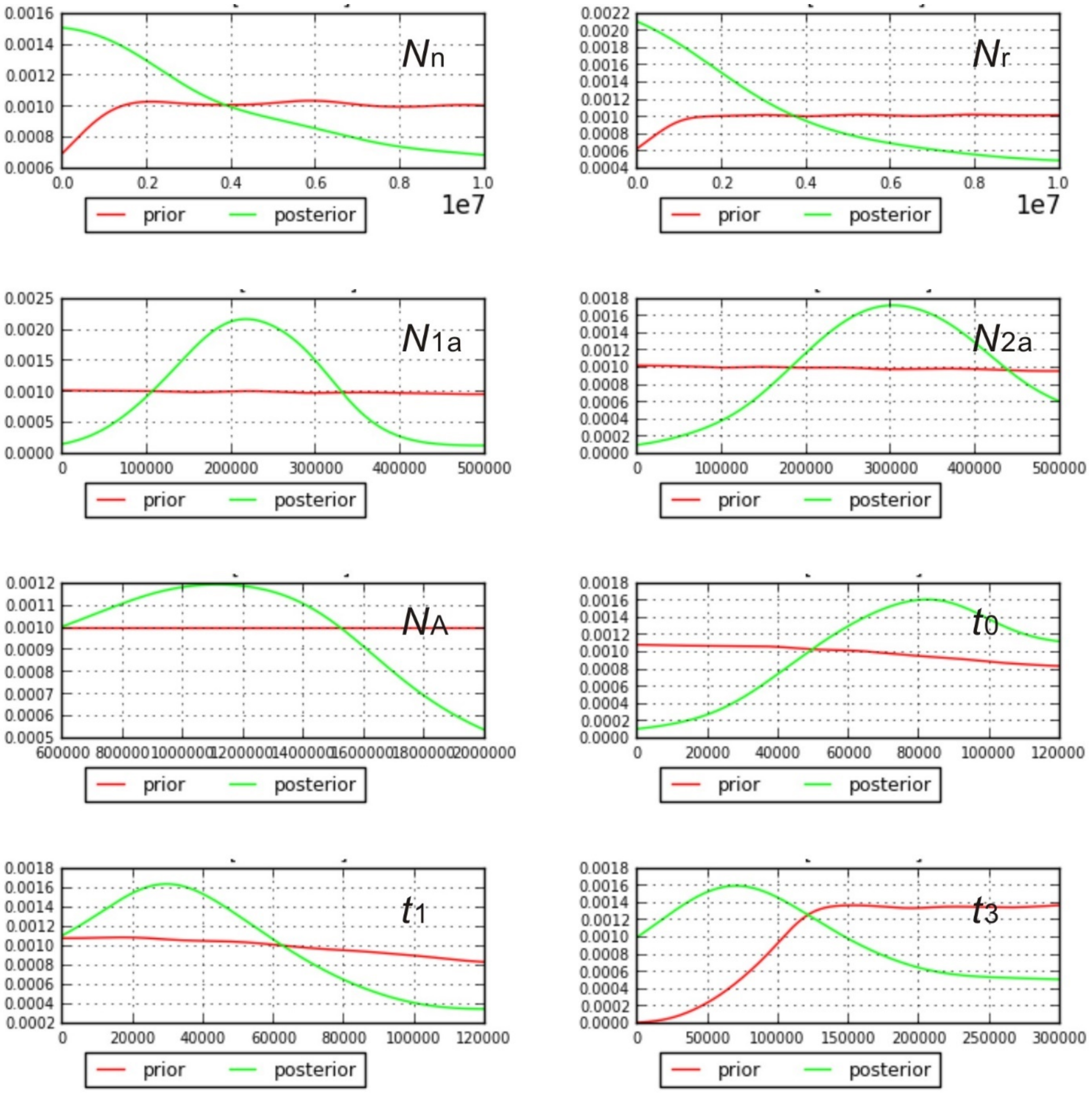

Figure S4. Prior and posterior distributions (Scenario 4 in Figure 1) of demographic parameters for *T. nucifera* var. *nucifera* (n) and *T. nucifera* var. *radicans* (r) estimated using DIYABC.

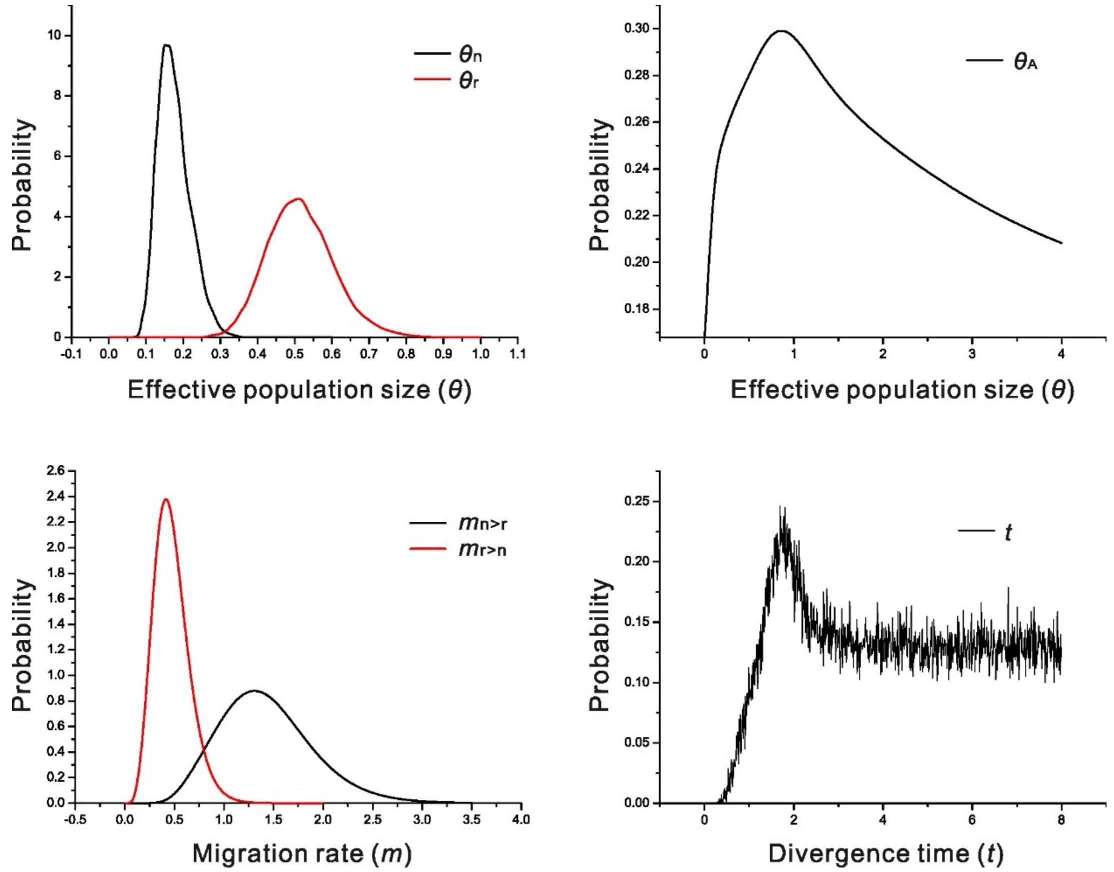

Figure S5. Posterior probability distributions of effective population size (*θ*), migration rate (*m*) and divergence time (*t*) between *T. nucifera* var. *nucifera* (n) and *T. nucifera* var. *radicans* (r) estimated using IM model based on 14 nuclear loci.

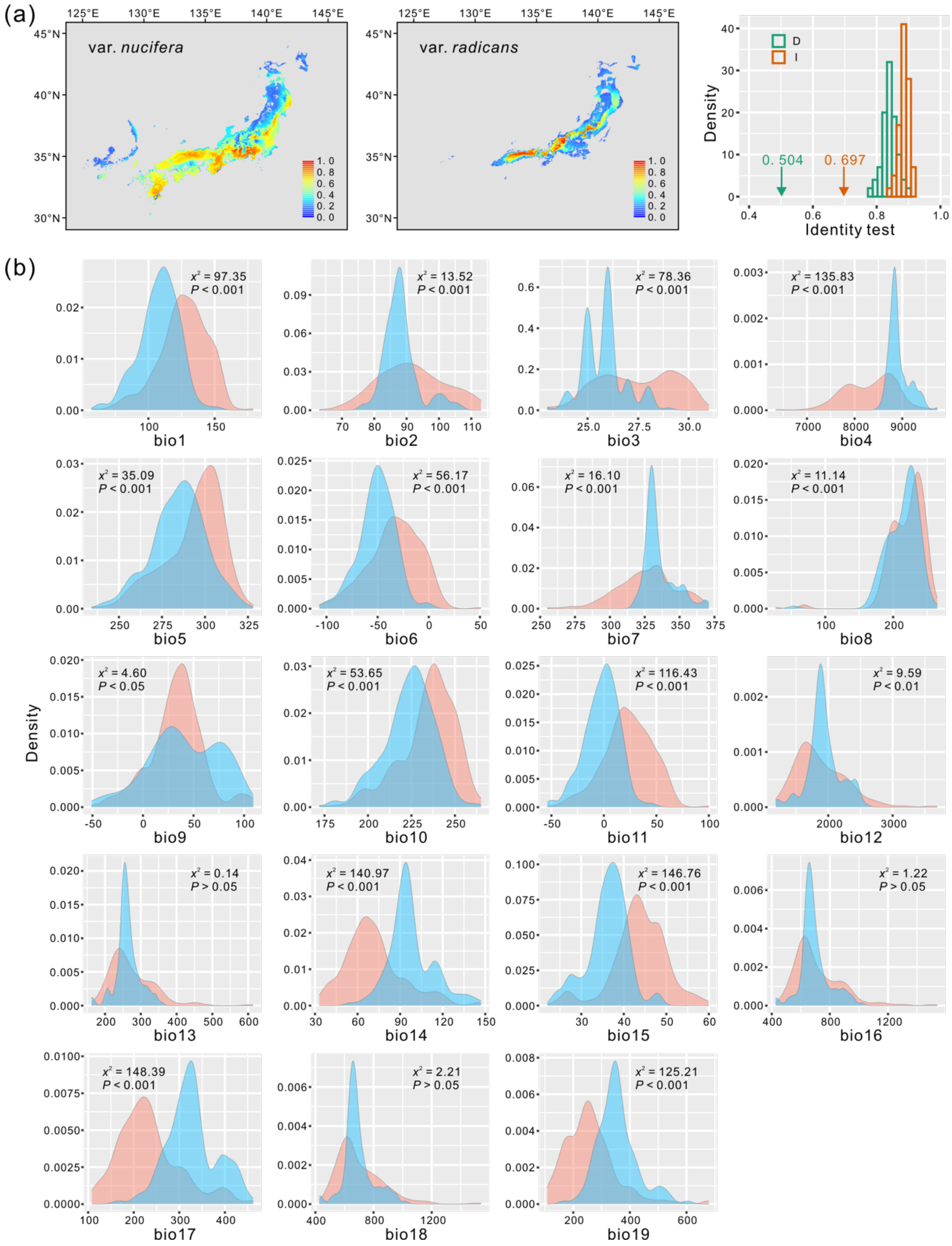

Figure S6. Climatic niche differentiation between *T. nucifera* var. *nucifera* and *T. nucifera* var. *radicans* in the Mid-Holocene (MH). (a) Climatic niches modeled using MAXENT and niche differentiation measured by identity tests (*I* and *D*) in ENMTools. (b) Kernel density plots of 19 climatic variables for *T. nucifera* var. *nucifera* (red curves) and *T. nucifera* var. *radicans* (blue curves). The differences of each ecological variable were assessed using nonparametric Kruskal-Wallis test with χ^2 and *P* value.

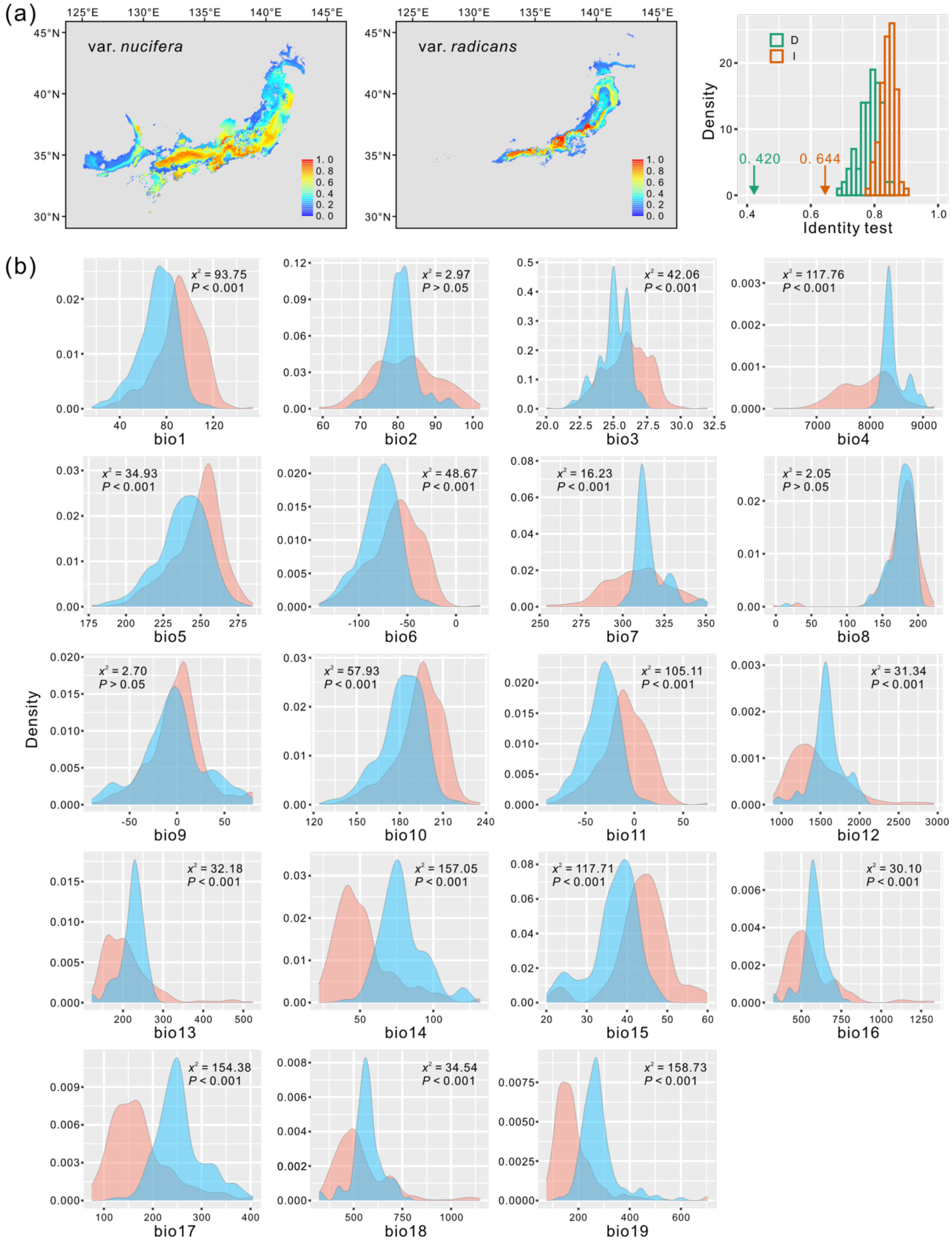

Figure S7. Climatic niche differentiation between *T. nucifera* var. *nucifera* and *T. nucifera* var. *radicans* in the Last Glacial Maximum (LGM). (a) Climatic niches modeled using MAXENT and niche differentiation measured by identity tests (*I* and *D*) in ENMTools. (b) Kernel density plots of 19 climatic variables for *T. nucifera* var. *nucifera* (red curves) and *T. nucifera* var. *radicans* (blue curves). The differences of each ecological variable were assessed using nonparametric Kruskal-Wallis test with χ^2 and *P* value.

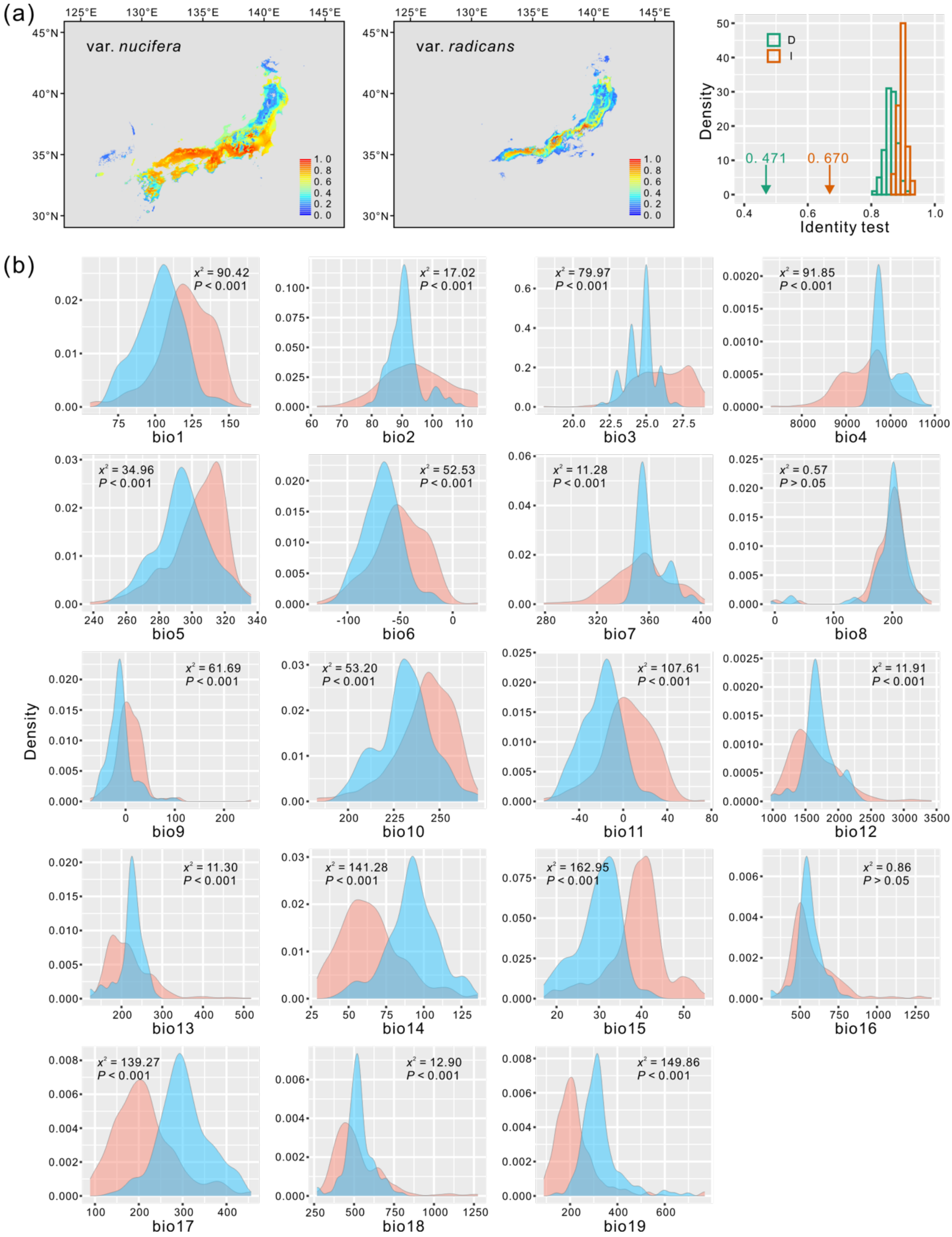

Figure S8. Climatic niche differentiation between *T. nucifera* var. *nucifera* and *T. nucifera* var. *radicans* in the Last Interglacial (LIG). (a) Climatic niches modeled using MAXENT and niche differentiation measured by identity tests (*I* and *D*) in ENMTools. (b) Kernel density plots of 19 climatic variables for *T. nucifera* var. *nucifera* (red curves) and *T. nucifera* var. *radicans* (blue curves). The differences of each ecological variable were assessed using nonparametric Kruskal-Wallis test with χ^2 and *P* value.
